# The Immune Microenvironment in Head and Neck Squamous Cell Carcinoma: on Subsets and Subsites

**DOI:** 10.1007/s11912-020-00938-3

**Published:** 2020-06-29

**Authors:** Niels E. Wondergem, Irene H. Nauta, Tara Muijlwijk, C. René Leemans, Rieneke van de Ven

**Affiliations:** Department of Otolaryngology-Head and Neck Surgery, Cancer Center Amsterdam, Amsterdam UMC, VU University medical center, De Boelelaan, 1117 Amsterdam, The Netherlands

**Keywords:** Head and neck squamous cell carcinoma, Tumor microenvironment, Innate and adaptive immune system, Tumor infiltrating lymphocytes, Prognosis, HNSCC subsites

## Abstract

**Purpose:**

To understand why some patients respond to immunotherapy but many do not, a clear picture of the tumor microenvironment (TME) of head and neck squamous cell carcinoma (HNSCC) is key. Here we review the current understanding on the immune composition per HNSCC subsite, the importance of the tumor’s etiology and the prognostic power of specific immune cells.

**Recent Findings:**

Large cohort data are mostly based on deconvolution of transcriptional databases. Studies focusing on infiltrate localization often entail small cohorts, a mixture of HNSCC subsites, or focus on a single immune marker rather than the interaction between cells within the TME.

**Summary:**

Conclusions on the prognostic impact of specific immune cells in HNSCC are hampered by the use of heterogeneous or small cohorts. To move forward, the field should focus on deciphering the immune composition per HNSCC subsite, in powered cohorts and considering the molecular diversity in this disease.

## Introduction

Head and neck squamous cell carcinoma (HNSCC) annually affects more than 700,000 patients globally, leading to over 350,000 deaths in 2018 [1]. It arises in the mucosal linings of the upper aerodigestive tract including the oral cavity, oropharynx, hypopharynx, and larynx. The most important risk factors for HNSCC are the use of tobacco, excessive alcohol consumption, and persistent infection with high-risk human papillomavirus (HPV) [2]. Despite aggressive and toxic treatment regimens including (a combination of) surgery, chemotherapy, and radiotherapy the 5-year overall survival (OS) remains a mere 40–50% and has seen little improvement in the past decades [3]. The effect of the recent addition of immune checkpoint inhibitors to the clinicians’ arsenal for recurrent and metastatic HNSCC on survival is therefore highly anticipated [4].

Treatment response and tumor progression are influenced by the interaction between the tumor and its surroundings, the tumor microenvironment (TME). Rather than considering the tumor as a group of malignant cells, the TME represents a complex eco-system in which the tumor and other constituents of the TME, such as T cells, B cells, natural killer (NK) cells, myeloid derived suppressor cells (MDSC), macrophages, dendritic cells (DC), and cancer associated fibroblasts (CAF), interact with each other. Tumor infiltrating lymphocytes (TILs) are considered the most crucial effectors of the host anti-tumor immune response, and their presence has been linked to improved survival in several cancer types accordingly [5, 6]. However, tumors have developed several mechanisms to escape the host immune response, including downregulation of HLA class I expression to avoid T cell recognition [7], induction of T cell apoptosis [8], recruitment of immunosuppressive cells such as regulatory T cells (Tregs), MDSCs, or M2 macrophages [9], inactivation of the antigen processing machinery preventing processing and presentation of tumor-associated antigens [9], and upregulation of checkpoint inhibitory molecules [10]. Additionally, tumor cells are under selective pressure through a dynamic process known as immunoediting, in which less immunogenic tumor cells are positively selected for their ability to escape the immune system and thus gain a survival advantage [11].

In recent years, the tumor immune microenvironment has gained much interest, especially in light of the recent advances in immunotherapy. The notion that the vast majority of HNSCCs are not destroyed upon anti-PD-1 checkpoint inhibition suggests that other immune suppressive mechanisms might be at play. In this review, we outline the various players in the TME of HNSCC and evaluate their function and prognostic significance. In addition, we discuss the current data available linking molecular alterations in HNSCC to the immune composition within the TME. Since HNSCC can be divided into HPV-related (mainly oropharyngeal squamous cell carcinomas (OPSCCs)) and HPV-unrelated (i.e., smoking- and alcohol-related) disease, it can be expected that the TME of both disease entities may vary. HPV-related HNSCCs are appreciated as a separate type of cancers since they differ significantly with respect to genomic and molecular aspects, clinical outcome, and immune microenvironment [12•, 13, 14]. In general, HPV-related HNSCC exhibit increased immune infiltrate compared with HPV-unrelated tumors [15, 16, 17•, 18]. Published studies where HPV status is not considered should therefore be interpreted with caution.

## Innate Immune Effector Cells

Innate effector cells, such as NK cells and neutrophils are seen as the first responders in case of tissue damage. Their functionality in the context of cancer is more often studied in the peripheral blood rather than at the tumor site. Relevant existing data on neutrophil- and NK cell presence in the TME is outlined below.

### Neutrophils

Neutrophils, or their precursors, can be attracted by developing tumor cells that secrete factors like IL-8, CCL4, or CCL5. Tumor-associated neutrophils (TAN) can be polarized towards an anti-tumor N1 phenotype and a pro-tumor N2 phenotype, dependent on the growth factors present within the TME [19]. N2-type TAN can promote tumor growth by supporting genetic instability, angiogenesis, cancer metastasis, and immune suppression [19, 20]. Regarding the presence of neutrophils in HNSCC, Trellakis et al. studied infiltration of polymorphonuclear granulocytes (PMNs) in tumors in the oropharynx (*n* = 71) and hypopharynx (*n* = 28) by staining for cells expressing the granulocytic marker CD66b or the azurophilic granule marker myeloperoxidase (MPO) [21]. The majority of the patients had stage IV disease (78%). PMN infiltration was observed in 93% of the studied cases, either in the tumor or the stroma. T4 tumors were found to be more highly infiltrated by PMN than lower T stage tumors. In a selection of advanced disease patients (*n* = 40) medium or strong PMN infiltration was a negative prognostic factor for OS in multivariate Cox regression analysis (*p* = 0.048). Dumitru et al. found a similar reduced survival in patients with oro- or hypopharyngeal cancer with high neutrophil infiltration and especially patients with multiple nodal metastasis had high rates of TAN [22]. The most unfavorable outcome was observed in this study when neutrophil infiltration was combined with expression of the actin-binding protein cortactin, suggestive of an interaction between TAN and cortactin in the TME promoting metastatic spread [22]. Reports on the quantification of neutrophils at other HNSCC subsites are lacking to date.

### NK Cells

NK cells can have direct cytolytic effect by producing factors like perforins and granzymes and they can secrete interferon-γ (IFN-γ), which promotes activation of myeloid cells and T helper-1 (Th1) cells. They can also promote tumor cell apoptosis via FasL or TNF-related apoptosis-inducing ligand (TRAIL). Additionally, NK cells can kill antibody-bound tumor cells through antibody-dependent cellular cytotoxicity (ADCC). Considering the latter, it is quite surprising that little is known on the presence of NK cells within the TME, since the immunological working-mechanism of the epidermal growth factor receptor (EGFR)-targeted antibody Cetuximab, used in the treatment of HNSCC, relies on NK-cell mediated ADCC [23]. In The Cancer Genome Atlas (TCGA) analyses (*n* = 500 HNSCC cases), HNSCC ranked third highest for the expression of NCR1 mRNA, which encodes the NK-specific marker NKp64 [24]. Patients with high NCR1 expression had a significantly better OS (*p* = 0.016). Looking at presented flow data for tumor-infiltrating leukocytes (TIL) in two HNSCC patients (from a cohort of *n* = 6), NK cells seem only a minor cell fraction within the viable TIL gate in these patients (0.45% or 0.98% of viable TIL, compared to 65% and 54% CD3^+^ T-cells, respectively). Wagner et al. quantified CD56^+^ NK cells in OPSCC (*n* = 140: 34 HPV-related, 106 HPV-unrelated) and found significantly higher NK cell density in HPV-related tumors and adjacent stroma compared to HPV-unrelated tumors (*p* = 0.004) [25]. NK cells were more abundant in the stroma compared to the tumor area. Co-staining with granzyme B and CD16 suggested the infiltrating NK cells to mainly represent cytotoxic NK cells, although potentially regulatory NK cells, lacking these markers, were detected and this was seen more often in HPV-unrelated tumors than in HPV-related tumors. In univariate Kaplan-Meier analyses, presence of CD56^+^ NK cells in either the tumor and/or stroma was linked to a better survival, both in HPV-related and HPV-unrelated disease. Mandal et al. also reported improved survival of HNSCC patients with high NK cell infiltration based on TCGA data [16].

## Myeloid Cells

### Dendritic Cells

DCs are antigen-presenting cells (APCs) which link the innate with the adaptive immune system. They pick up antigens at the site of the tumor and, when properly activated, are able to induce a tumor-specific T cell response. Early studies already suggest that a greater number of myeloid DCs (mDCs) is associated with increased TILs, lower rate of metastases, less recurrence, and improved survival in patients with HNSCC [26, 27]. Recent studies confirm this positive correlation [28–32]. This implies that DCs play a pivotal role in the immunosurveillance of the host against HNSCC. Contrary, several studies did not replicate the link between DC density and clinical parameters [33–37]. Collectively, there is no consistency concerning the prognostic significance of DC density for HNSCC. This may be explained by small study sizes, discrepancy concerning the type of DC markers, and heterogeneous patient populations. By way of illustration, tumor subsite, which has significant impact on the immune landscape [12•, 13, 14], varied within and between aforementioned studies. Moreover, HPV status was not always included. HPV E6 and E7 may impede with macrophage inflammatory protein 3 (MIP-3) transcription and E-cadherin levels leading to reduced DC activity [38, 39]. In studies where HPV status was included, some found elevated DC numbers in HPV-related HNSCC [12•, 15], while others observed no differences [17•, 40]. Notably, Kindt et al. detected significantly lower DCs in HPV-related compared with HPV-unrelated tumors [14]. The prognostic value of DCs when including HPV status is still poorly studied. Nguyen et al. could not find a significant correlation between DC levels and survival after controlling for among others HPV status [12•]. In addition, Kindt et al. observed no correlation between the number of DCs and survival of HPV-related HNSCCs. However, the DC number was significantly associated with increased recurrence free survival and OS in HPV-unrelated disease [14]. Taken together, the prognostic relevance of mDCs in HNSCC remains unresolved.

Besides mDCs, plasmacytoid DCs (pDCs) infiltrate the TME of HNSCC [34, 41–43]. At steady state, pDCs circulate in the blood and can be found in lymphoid organs. Upon infection, pDCs infiltrate peripheral tissues [44]. Within the TME, pDCs predominately reside within the connective tissue, close to the tumor [34, 41–43]. pDCs are able to elicit an anti-tumor response by secreting high levels interferon-α (IFN-α) upon triggering of the Toll-like receptor 9 (TLR9) [44]. However, it appears that pDCs display diminished IFN-α production in HNSCC [41, 42]. Also, tumors are able to downregulate TLR-9 on pDCs [41], indicating that although pDCs infiltrate the TME, their ability to elicit an anti-tumor response is diminished. Moreover, it has been suggested that pDCs in the absence of appropriate stimulation promote tolerance by inducing Tregs [41, 45]. In concordance with this, enhanced pDC numbers significantly associate with tumor size, lymph node metastases, and poor clinical outcome in HNSCC [34, 42]. Han et al. demonstrated that infiltrating pDCs are an independent prognostic factor. They also examined whether the number of pDCs differed upon HPV-status but found no correlation between pDCs and HPV infection [42]. Contrary, Partlová et al. observed more pDCs in HPV-related HNSCC, but this difference was not significant [15]. Additional studies are required to understand the prognostic value of pDCs at different HNSCC subsites and in relation to HPV-status.

### Macrophages

Macrophages are found in abundance within the TME and appear in different phenotypes. Macrophages activated by IFN-γ polarize into a M1 phenotype and contribute to anti-tumor immune responses, as opposed to M2 macrophages, driven by interleukin (IL)-4, which are characterized by stimulating anti-inflammatory and pro-tumoral responses [46–48]. It has become evident that the density of CD68^+^ cells, a general marker for macrophages, is elevated in HNSCC compared with normal mucosa [49–54]. CD68^+^ tumor–associated macrophages (TAMs), defined as macrophages located in or close by the tumor, were found to correlate with lymph node metastases and poor survival in HNSCC [49–52, 54–58]. This correlation was not observed in all studies [12•, 59–62]. Of note, the expression of CD163, a specific M2 marker [63], may be prognostically more informative. HNSCC cells drive TAMs towards M2 polarization [64]. In turn, M2 TAMs contribute to migration and invasion of HNSCC cells [65, 66]. Accordingly, two studies found no association between CD68 positivity and clinical outcome while increased CD163^+^ TAMs were an independent prognostic factor [61, 62]. The prognostic value of M2 TAMs was confirmed by several other studies [58, 67–69], though not in all [32, 70].

The ratio of M1/M2 TAMs indicates a better prognosis in both HPV-related and HPV-unrelated HNSCC, and it has been reported that HPV-related tumors have a higher M1/M2 ratio [17•]. Furthermore, lower M2/CD68^+^ TAM ratios have been observed in HPV-related HNSCC compared with HPV-unrelated [12•, 52, 60, 71, 72]. In line with this, HPV-unrelated tumors display augmented M2 infiltration [73]. While aforementioned studies show consistency, several studies could not replicate these differences in CD68 and/or CD163 expression between HPV-unrelated and HPV-related tumors [53, 59, 69, 70, 74]. These discrepant results may be explained by variation in tumor subsites, treatment, or cutoff values concerning marker expression. In summary, compelling evidence suggests a role for M2 TAMs in the progression of HNSCC. HPV-related tumors seem to be more enriched for M1 TAMs, while HPV-unrelated tumors appear to be more enriched for M2 TAMs.

### Myeloid-Derived Suppressor Cells

MDSCs are characterized by their ability to inhibit the innate as well as adaptive immune system. They suppress CD4^+^ and CD8^+^ T cells, induce Tregs, and act together with macrophages resulting in a shift towards an immunosuppressive phenotype by elevating levels of IL-10 and decreasing IL-12 [75–77]. Elevated levels of MDSCs in HNSCC compared to normal mucosa have been described [53, 74, 78, 79]. Moreover, increased accumulation of MDSCs correlates with high clinical stage and pathological grade [79]. It remains elusive whether the infiltration of MDSCs differs upon HPV status. Although three studies concluded that MDSC levels do not differ between HPV-related and HPV-unrelated HNSCC [53, 69, 74], additional studies are required to confirm this.

## Adaptive Immune Cells

### CD8^+^ Effector T Cells

After activation by antigenic and cytokine stimulation by APCs like DCs, naïve CD8^+^ T cells differentiate into either memory or cytotoxic effector T cells [80]. Cytotoxic T cells constitute a subset of T cells with the ability to recognize and kill tumor cells and therefore serve as central players within the anti-tumor response. Indeed many studies report a positive correlation of higher CD8^+^ TIL with improved survival in HNSCC patients [32, 81–85]. However, the effect seems to be affected by tumor subsite, relative amount to other tumor infiltrating immune cells, spatial distribution, and HPV status.

In OCSCC, a prognostic benefit of high CD8^+^ TIL has been described [85–89, 90•], but the majority of reports show no significant association of high CD8^+^ TIL with OS or DSS [56, 91–96]. This lack of consistency might in part be explained by the relative amounts of CD8^+^ TILs to other TILs affecting their function. For instance, a high CD8^+^/CD4^+^ ratio confers superior DSS and DFS [97], and a low CD8^+^/forkhead box protein 3 (FoxP3) ratio confers inferior OS and DFS [98, 99]. Interestingly, two recent meta-analyses on the role of CD8^+^ T cells in OCSCC showed contradictory results. This could be due to the low amount of studies that qualified for meta-analysis, especially by Hadler-Olsen et al. [100, 101].

In OPSCC, the prognostic role of CD8^+^ TILs seems more pronounced with a substantial amount of papers reporting a positive effect on survival of high CD8^+^ TIL both stromal and intratumoral [59, 85, 102–108]. HPV-related OPSCC show a significantly higher infiltration of CD8^+^ T cells [12•, 15, 70, 74, 82, 85, 104–106]. In fact, it has been reported that HPV-related OPSCC patients with a low CD8^+^ TIL count do not show the typical improved survival associated with HPV-related OPSCC [109]. This could point to a role for the local immune response in the survival of HPV-related OPSCC patients. Not all studies were able to find this association, however [110]. Although investigated to a lesser extent, high CD8^+^ TIL counts have been associated with survival benefit in laryngeal squamous cell carcinoma [85, 111, 112].

### CD4^+^ T Cells

CD4^+^ T cells represent a heterogeneous cell type which can be sub-classified into various subtypes including Th1, Th2, Th9, Th17, follicular helper T cells (Tfh), and Tregs [113]. This hampers the evaluation of their role in HNSCC as observed effects could be attributable to any of these subtypes, and staining for CD4 alone might not be sufficient. This could explain the ambiguous role of CD4^+^ T cells in HNSCC: some papers report a favorable prognostic effect of higher CD4^+^ TIL [12•, 85, 114], while others report the opposite [88, 115]. However, no significant association between CD4^+^ TIL and survival has been reported most frequently [81, 86, 91, 94, 96, 102]. Although in their meta-analyses, de Ruiter et al. did find a prognostic benefit of higher CD4^+^ T cell infiltration in HNSCC, the authors stress to interpret this with caution given the paucity of papers eligible for analyses and a high suspicion of publication bias [84]. In two recent meta-analyses for OCSCC specifically, Huang et al. report no prognostic value [100], whereas Hadler-Olsen et al. found data to be insufficient for analyses [101]. Recently, Cillo et al. [116•] reported on scRNAseq comparing HPV-related and HPV-unrelated HNSCC, and showed that HPV-related tumors are enriched in the presence of a Tfh cell population when compared with HPV-unrelated tumors. In TCGA data, a Tfh-high profile corresponded with improved survival [116•]. It must be mentioned that while the majority of HPV-related tumors in this study were OPSCC, the HPV-unrelated tumors were a mix of OCSCC, OPSCC, larynx, and hypopharynx. In all, the role of CD4^+^ T cells in HNSCC remains to be clarified. Investigation of CD4^+^ T cell subpopulations at various HNSCC subsites specifically is warranted.

### Regulatory T Cells

Tregs are a subpopulation of CD4^+^ T cells which can be identified by the expression of the transcription factor FoxP3 [113]. They play a critical role in maintaining host-tolerance and thus preventing auto-immunity, by regulating other immune cells including DCs, NK cells, B-cells, CD4^+^ and CD8^+^ T cells, but in doing so also facilitate tumor immune escape and tumor progression [117]. Indeed a large meta-analysis on the prognostic value of FoxP3 showed that high Treg infiltration is associated with inferior OS in several tumor types, but not in HNSCC [118]. In fact, when considering HNSCC in general, higher CD4^+^FoxP3^+^ T cell infiltration correlates with better survival [30, 119, 120]. This does however not seem to apply to all HNSCC subsites as for OCSCC most studies do report lower CD4^+^FoxP3^+^ TIL counts to be related with improved survival [121–124]. Only one study found an association in the opposite direction [97], but most, including two meta-analyses, failed to show a significant correlation of FoxP3 with survival in OCSCC [89, 92, 100, 101, 125].

Echarti et al. recently reported high stromal numbers of Tregs to be correlated with improved survival in HNSCC, and epithelial numbers of Tregs to only gain prognostic importance when stratifying for “inflamed”, “immune excluded”, and “immune desert” tumors based on total number of infiltrating CD8^+^ T cells [126]. This might in fact represent the CD8^+^/FoxP3^+^ ratio, which confers survival benefit when lower in OCSCC, as published previously by Chen et al. and more recently by Ni et al. [99, 124]. Interestingly, the intracellular localization of FoxP3 might play a role, as a shift in the subcellular localization of FoxP3 expression from cytoplasmic to nuclear has been described after TCR/CD28 activation [127, 128]. This indicates that a subgroup of FoxP3^+^ T cells might in fact have an effector function. Weed et al. have found that in OCSCC, cytoplasmic localization of FoxP3 was associated with lower risk of recurrence, as opposed to nuclear localization [129]. In addition, Feng et al. report the spatial relation of FoxP3^+^ T cells to CD8^+^ T cells to affect OS in OCSCC. In their study using multispectral imaging on 119 HPV-unrelated OCSCC, they showed that a higher density of FoxP3^+^ T cells within 30 μm of CD8^+^ T cells was significantly associated with worse OS [90•].

In OPSCC, a positive correlation of higher CD4^+^FoxP3^+^ T cell infiltration with improved survival is reported [59, 85, 120, 130, 131], but a significant association with poor survival has also been described [132]. When considering HPV, it seems that papers reporting significantly higher CD4^+^FoxP3^+^ infiltration in HPV-related OPSCC [103, 130, 131, 133] are in balance with reports that do not find such relation [104, 106, 120, 134, 135]. In their study, Ward et al. reported higher absolute counts of FoxP3^+^ T cells in HPV-related OPSCC and correlated this to improved survival [109]. However, the authors state the observed absolute increase could merely be a reflection of higher overall T cell infiltration in HPV-related OPSCC, since relative amounts of FoxP3 did not differ significantly. One other study described such correlation [131]. In their meta-analysis, de Ruiter et al. were unable to analyze HPV-related tumors due to insufficient data [84].

### B Lymphocytes

The composition and prognostic role of B-cells in the TME of HNSCC is relatively understudied. As part of the adaptive immune system, B lymphocytes play an important role in the immune response following the onset of malignant tumors [136]. Mature B cells can produce antibodies which could bind to tumor cells and induce ADCC by NK cells or Fc-receptor mediated phagocytosis by macrophages, they can also act as APCs or can directly interact with CD4^+^ T cells (through CD40/CD40L) or CD8^+^ T cells (through CD27/CD70), thereby, providing help to the anti-tumor immune response [137]. In addition to these anti-tumor features, regulatory B-cells (Bregs) are thought to stimulate tumor growth by negatively interacting with other immune cells as well as with tumor cells. It is assumed that Bregs inhibit NK cell- and cytotoxic T cell–mediated tumor immunity, suppress the differentiation of Th1/Th17 cells, and promote the alteration of T-helper cells and macrophages towards the Th2/M2 types. Moreover, Bregs might disable the cellular immune response against the tumor by promoting the conversion of CD4^+^/CD25^−^ T lymphocytes to FoxP3^+^ Tregs, thereby, enabling tumor growth and the development of metastasis. Bregs secrete IL-6, IL-10, IL-35, and TGF-β, all known for their suppressive effect on the immune response [138].

HPV-related HNSCC were shown to contain increased percentages of tumor-infiltrating CD19^+^/CD20^+^ B lymphocytes compared with HPV-unrelated HNSCC and non-cancerous mucosa [17•, 70, 74, 139•, 140, 141]. When performing RNA-sequencing, it was even shown that phenotypic differences exist between B lymphocytes in HPV-related and HPV-unrelated HNSCC [140]. Hladíková et al. found that in HPV-related OPSCCs, the B cell population within the TME seems to be represented mainly by memory B lymphocytes with an activated, antigen-experienced phenotype, characterized by high expression of CD27, no expression of IgD and low expression of IgM [141]. When they subsequently divided the HPV-related OPSCCs into two groups based on the amount of B cell infiltrate (B^low^ (B cell proportions < 0.5% of total cells) versus B^hi^ tumors), they noticed that in the B^hi^ group the tumor-infiltrating B cells displayed significantly higher levels of activation markers like HLA-ABC, HLA-DR, CD86, and CD40 when compared with the B^lo^ group, corresponding with an activated phenotype. Moreover, B^hi^ OPSCCs possessed higher proportions of proliferating Ki-67^+^ B cells than the B^low^ counterparts [141].

The prognostic impact of B lymphocytes in the TME of HNSCC remains somewhat ambiguous. While most studies report a favorable effect of B lymphocytes on HNSCC patient survival [17, 141–143], some studies fail to find any effect on survival [70] or even claim that certain subsets of B cells, particularly Bregs, may contribute to an immunosuppressive, cancer promoting microenvironment [144]. Distel et al. demonstrated that the prognostic impact of CD20^+^ B cells may be dependent on the type of treatment and stage of disease [145]. In their study, they investigated 115 patients with oro- or hypopharyngeal squamous cell carcinoma by dividing them in two groups: a low-risk group (*n* = 62) of early stage disease treated with primary surgery and postoperative radiotherapy, and a high-risk group (*n* = 53) of inoperable, advanced stage disease treated with definitive chemoradiotherapy. Immunohistochemistry for CD3, CD4, CD8, CD20, CD68, FoxP3, and granzyme B was performed on all pretreatment biopsies and percentages of the various subsets of TIL were correlated with survival. Surprisingly, it was observed that in the low-risk group higher numbers of CD20^+^ B cells were consistently associated with improved locoregional tumor control (*p* = 0.02), while it was negatively associated with prognosis in the high-risk group (*p* = 0.04). Of note, B cells have the ability to form tertiary lymphoid structures (TLS) when they aggregate in a network of follicular DCs surrounded by T cells and high-endothelial venules [146]. In OCSCC, TLS are more frequently seen in stage I–II than in stage III–IV disease, and the presence of TLS is associated with improved survival [147]. In their scRNAseq study, Cillo et al. reported an increased presence of germinal center B cells in HPV-related versus HPV-unrelated HNSCC, corresponding with the presence of TLS [116•].

## Stroma

### Cancer-Associated Fibroblasts

The most abundant, non-immune, cell type recognized in stroma are cancer-associated fibroblasts (CAFs). Without any doubt, CAFs promote tumor development by supporting tumor cell proliferation, invasion, and metastasis. The presence of CAFs within the TME often is linked with stromal desmoplasia through deposition of collagen. The most commonly used markers to identify CAFs are α-smooth muscle actin (α-SMA), integrin α6, and fibroblast activation protein (FAP). In addition to producing growth factors like epidermal growth factor and vascular endothelial growth factor, CAFs produce matrix metalloproteinases that aid in the remodeling of the extracellular matrix and facilitate tumor outgrowth and metastasis. While much is known about the detrimental factors that are produced by CAFs, the exact origin of the CAFs within the TME remains quite elusive, as they have been described to derive from various different cell types [148]. CAFs promote immune suppression in the stromal compartment by producing high levels of TGF-β, IL-10, and IL-6, while at the same time recruiting many inflammatory cells through secreted chemokines [149].

In OCSCC in the tongue, the presence of CAFs, as identified by IHC, was found to be an independent prognostic factor, negatively impacting OS and DFS [150]. Similarly, CAF-related gene expression was linked to worse prognosis in OCSSC [151].

Multiple studies have looked at the effects of isolated CAFs and the factors they produce on the growth and invasion capacities of HNSCC cell lines [152, 153]. While there is a general consensus that CAFs are a negative prognostic factor in HNSCC, surprisingly few studies can be found that actually tested this in (large) cohorts of patients by quantifying CAFs in the TME. The studies that are available primarily focus on the prognostic value of CAFs in OCSCC. Dhanda et al. stained 104 OCSCC for the CAF markers αSMA and SERPINE1 and found high expression to relate with poor clinical outcome and extracapsular spread [154]. In OPSCC (*n* = 44), the collagen proteins COL8A1 and COL11A1 were described to be expressed on tumor cells as well as CAFs in the TME. While a reduction in survival was mentioned, which did not reach statistical significance in this small group of patients, these data were not shown [155]. Puram et al. [156•] performed single-cell RNAseq on ~ 6000 cells isolated from OCSCC samples (*n* = 18) and found CAFs to be a large determinant of the mesenchymal molecular subgroup of HNSCC previously identified by the TCGA workgroup [157]. This molecular subgroup is known for its invasive character and poor survival outcome and is dominated by genes related to epithelial to mesenchymal transition (EMT). Puram et al. propose that the tumor cells in this subgroup are similar to those defined in the basal molecular subgroup, but that the presence of CAFs within the TME promotes the EMT profile. There seems to be a void to be filled, where CAFs are quantified in the different subsites of HNSCC combined with immune composition analysis, not at the transcriptional level, but studying the actual cell numbers and their spatial localization.

## Molecular Landscape and TME

The tumor mutational landscape and the TME are two of the major determinants of personalized medicine. To comprehensively characterize the TME, the effect of the mutational landscape on the TME should therefore be taken into consideration. We outline the few studies that have investigated their relation below.

Saloura et al. [73] studied the relation of genetic alterations to CD8^+^ T cell infiltration using both the TCGA and Chicago Head and Neck Genomics (CHGC) cohorts. Tumors were classified into high or low CD8^+^ T cell inflamed phenotype (TCIP-H vs TCIP-L) based on their chemokine signature. Results showed that TCIP-H tumors were enriched for mutations in CASP8, EP300, EPHA2, and HRAS and had more frequent amplifications of CD274, PDCD1LG2, JAK2, and KDM4C. TCIP-L tumors showed higher rates of NSD1 mutations, EGFR and YAP1 amplifications and CDKN2A deletion. Unfortunately, HPV-related tumors were too infrequent in the TCGA database to analyze the impact of HPV on the genetic landscape of TCIP-H and TCIP-L tumors. Mandal et al., also exploiting the TCGA database, showed that the proportion of mutational processes attributable to tobacco smoking inversely correlated with measures of immune infiltration, indicative of the immunosuppressive effect of tobacco smoking [16]. Although immunologically cold, these tumors were associated with high mutational loads. These tumors are expected to be more immunogenic due to higher neoantigen load [158], but in their study, Mandal et al. were unable to find any significant correlation of mutational load to measures of immune infiltration [16]. This was substantiated by Saloura et al. in their later study [73].

In a transcriptional study, linking TP53 mutational status in HNSCC with immune signatures, neutrophil, a NK cell, pDC, Treg, CD8+ TIL, and B cell gene signatures were reported to be significantly lower in TP53 mutated compared with TP53 wild-type tumors (*P* = 0.001) and higher in HRAS-mutated tumors (*p* = 0.033). [159]. For TP53 mutants, these subsets were lower both in HPV-related and HPV-unrelated tumors, though the significance was most pronounced between mutant and wild-type in HPV-related cases. Significantly fewer NK cells were reported to be present in TP53 mutant tumors compared with TP53 wild-type tumors (*p* = 6.4·10^−8^), and more NK cells were present in HRAS-mutated tumors than HRAS wild-type tumors (*p* = 0.012). Aligned with the data from Wagner et al. [25] NK cell–related genes were more abundant in HPV-related tumors than HPV-unrelated tumors [159].

All above analyses were performed on several multi-omics datasets, and HNSCC subsites were not specified.

## Conclusion and Future Directions

**Fig. 1:**
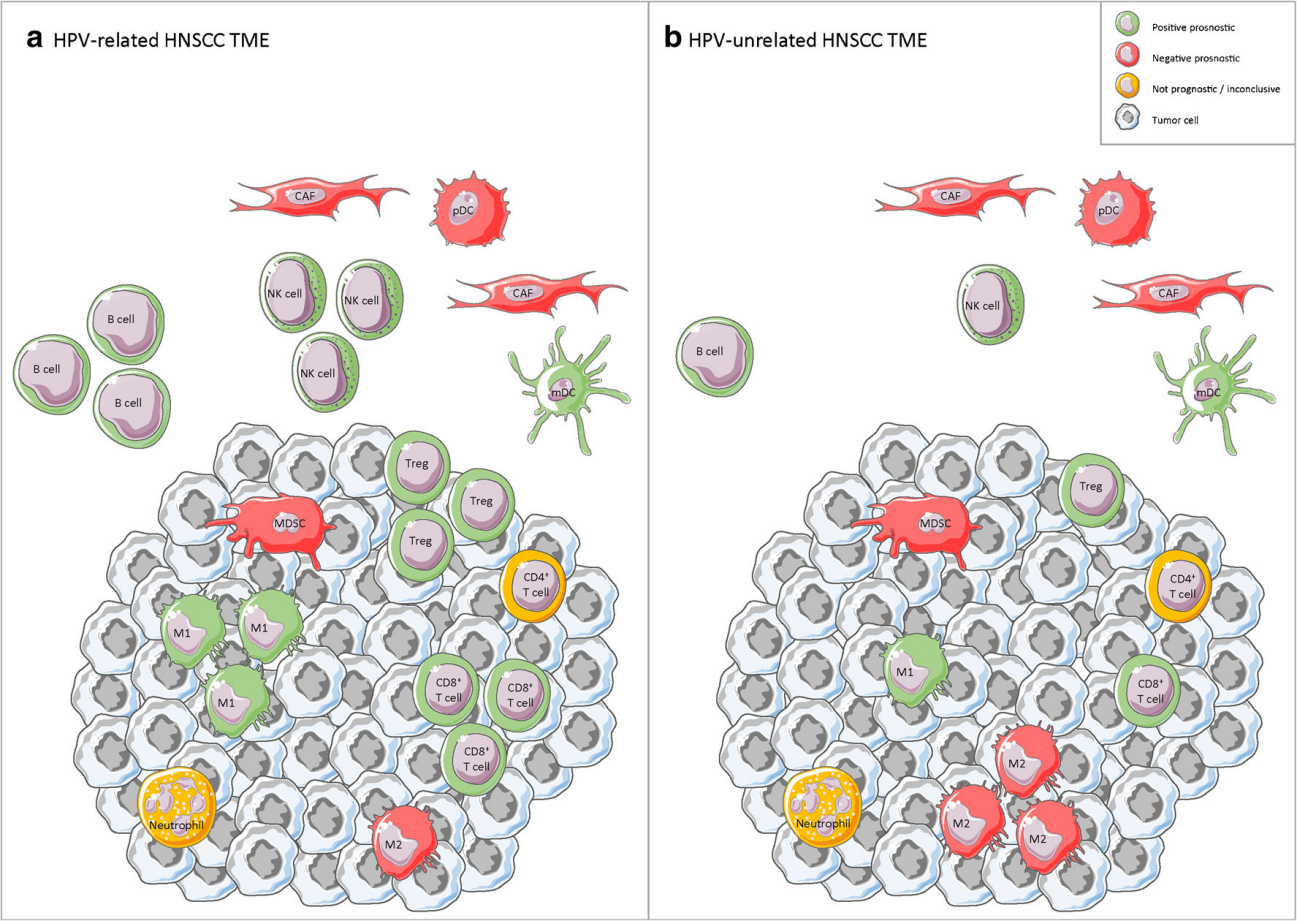
Schematic overview of the current understanding on the differences between the immune cells infiltrating HPV-related (**a**) and HPV-unrelated (**b**) HNSCC. Abbreviations: CAF: cancer associated fibroblast; pDC: plasmacytoid dendritic cell; mDC: myeloid dendritic cell; NK cell: natural killer cell; M1: M1 macrophage; M2: M2 macrophage

In Table 1, we have combined the observations from the studies discussed in this review to provide an overview of the current understanding of the prognostic value of different immune subsets at different HNSCC subsites. While it seems evident that immune cells infiltrating the TME in HNSCC have relevance with regard to the clinical prognosis of patients, there is a clear need for more extensive studies to fill in some gaps. Our opinion is that the field should focus more on subsite specific analyses, clearly separating HPV-related and HPV-unrelated HNSCC. The current understanding on the differences between the immune cells infiltrating HPV-related and HPV-unrelated HNSCC and their link to prognosis are visualized in Fig. 1. Also, rather than focusing on studies trying to mine the presence of immune cells from large omics datasets, understanding the spatial interaction between tumor cells, immune cells, and CAFs in the stromal compartment, is in our opinion crucial to getting a better understanding of the TME in HNSCC. This will hopefully result in clearer answers on the prognostic power of the immune infiltrate, as a whole or of specific cell subsets, and will aid in defining those patients most likely to respond to (immuno)therapy strategies as well as create new hypotheses as to how to improve the immune infiltrate in patients who seem to fit a less favorable profile.

**Table 1:**
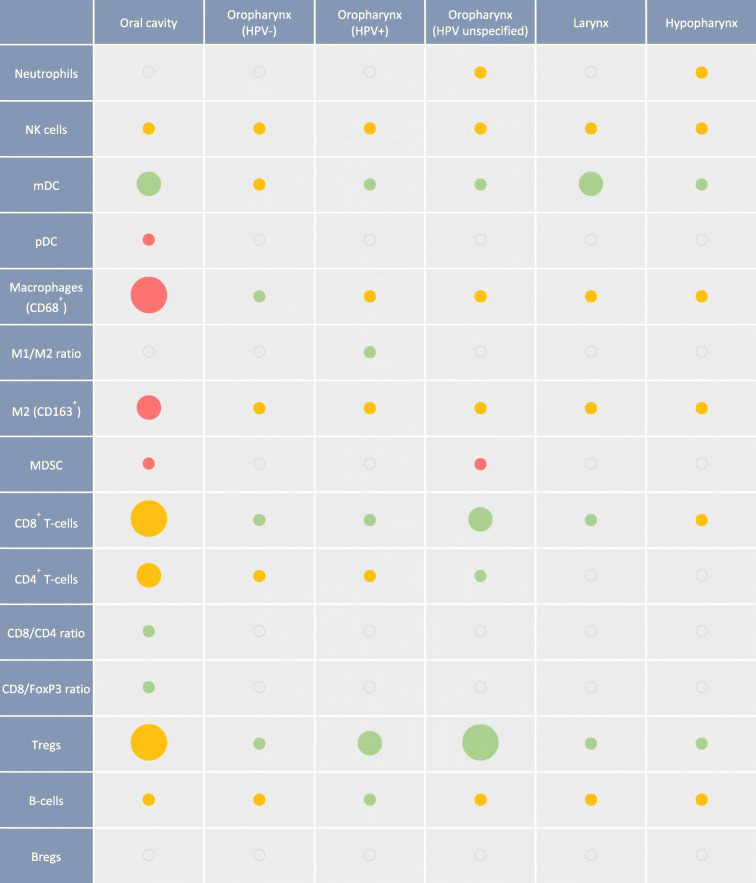
Overview of the current understanding of the prognostic value of different immune subsets at different HNSCC subsites. Green indicates > 50% of papers report positive correlation, red indicates > 50% of papers report negative correlation and yellow indicates > 50% of papers report no correlation, or number of studies reporting opposite effects are equal. Size represents amount of papers considered. Small 0–5 papers; medium 6–10 papers; large > 10 papers. Abbreviations: *HPV*, human papillomavirus; *NK cells*, natural killer cells; *mDC*, myeloid dendritic cells; *pDC*, plasmacytoid dendritic cells; *MDSC*, myeloid derived suppressor cells; *Tregs*, regulatory T-cells; *Bregs*, regulatory B-cells
